# [^18^F]Florbetapir PET/MR imaging to assess demyelination in multiple sclerosis

**DOI:** 10.1007/s00259-019-04533-y

**Published:** 2019-10-21

**Authors:** Antonio Carotenuto, Beniamino Giordano, George Dervenoulas, Heather Wilson, Mattia Veronese, Zachary Chappell, Sotirios Polychronis, Gennaro Pagano, Jane Mackewn, Federico E. Turkheimer, Steven C. R. Williams, Alexander Hammers, Eli Silber, Peter Brex, Marios Politis

**Affiliations:** 1 Neurodegeneration Imaging Group, Maurice Wohl Clinical Neuroscience Institute, IoPPN, King’s College London, 125 Coldharbour Lane, Camberwell, London, SE5 9NU UK; 2 Multiple sclerosis Clinical Care and Research Centre, Department of Neuroscience, Federico II University, Naples, Italy; 3 Department of Neuroimaging, Institute of Psychiatry, Psychology and Neuroscience, King’s College London, London, UK; 4 King’s College London & Guy’s and St Thomas’ PET Centre, School of Biomedical Engineering and Imaging Sciences, Faculty of Life Sciences and Medicine, King’s College London, St Thomas’ Hospital, London, UK; 5 Department of Neurology, King’s College Hospital NHS Foundation Trust, London, UK

**Keywords:** Multiple sclerosis, PET, [^18^F]florbetapir, Demyelination, Pathology

## Abstract

**Purpose:**

We evaluated myelin changes throughout the central nervous system in Multiple Sclerosis (MS) patients by using hybrid [^18^F]florbetapir PET-MR imaging.

**Methods:**

We included 18 relapsing-remitting MS patients and 12 healthy controls. Each subject performed a hybrid [^18^F]florbetapir PET-MR and both a clinical and cognitive assessment. [^18^F]florbetapir binding was measured as distribution volume ratio (DVR), through the Logan graphical reference method and the supervised cluster analysis to extract a reference region, and standard uptake value (SUV) in the 70–90 min interval after injection. The two quantification approaches were compared. We also evaluated changes in the measures derived from diffusion tensor imaging and arterial spin labeling.

**Results:**

[^18^F]florbetapir DVRs decreased from normal-appearing white matter to the centre of T2 lesion (*P* < 0.001), correlated with fractional anisotropy and with mean, axial and radial diffusivity within T2 lesions (*coeff.* = −0.15, *P* < 0.001, *coeff.* = −0.12, P < 0.001 and *coeff.* = −0.16, P < 0.001, respectively). Cerebral blood flow was reduced in white matter damaged areas compared to white matter in healthy controls (−10.9%, *P* = 0.005). SUV_70–90_ and DVR are equally able to discriminate between intact and damaged myelin (area under the curve 0.76 and 0.66, respectively; *P* = 0.26).

**Conclusion:**

Our findings demonstrate that [^18^F]florbetapir PET imaging can measure in-vivo myelin damage in patients with MS. Demyelination in MS is not restricted to lesions detected through conventional MRI but also involves the normal appearing white matter. Although longitudinal studies are needed, [^18^F]florbetapir PET imaging may have a role in clinical settings in the management of MS patients.

## Introduction

Multiple Sclerosis (MS) usually presents with episodes of transient neurological deficits, called ‘relapses’ [1]. Relapses mirror the occurrence of focal areas of acute demyelination within the brain, optic nerves and spinal cord [2]. Demyelination can cause axonal loss, which over time causes permanent disability [3]. Demyelination can be qualitatively detected as areas of high signal on T2-weighted magnetic resonance imaging (MRI) sequences or as low signal in T1-weighted spin-echo images on MRI scans [4]. Advanced MRI techniques might provide a better insight into multiple sclerosis pathology. In example, diffusion tensor imaging enables us to detect microstructural tissue damages in brain regions [5, 6], whereas arterial spin labelling provides a quantitative measure of the cerebral blood flow. However, conventional MRI, diffusion tensor imaging and arterial spin labelling only represent indirect measures of tissue modifications and lack specificity towards myelin integrity. Therefore, although previous studies have reported the association between brain microstructural changes and physical disability in MS, the effect of demyelination on physical disability might not be disentangled for other pathological factors such as axonal loss or gliosis for the low specificity of MRI techniques.

Over the past years, positron emission tomography (PET) imaging has become a promising modality to monitor the biological processes underlying MS pathology [7]. Radioligands developed to measure amyloid-β pathology in grey matter for Alzheimer’s disease, such as [^11^C]PIB, [^18^F]florbetaben and [^18^F]florbetapir [8–10] also showed affinity for myelin protein. This is due to the fact that these stilbene and benzothiazole derivatives have a very flat structure, interacting with the secondary structure of myelin basic protein similarly to the way they interact with amyloid [11, 12]. In fact, in healthy white matter, myelin basic protein has a particular two-dimensional secondary structure as it acts as a hinge between lipid bilayers. In MS, this structure is damaged which, consequently, leads to a decreased binding of amyloid-β PET tracers [13]. Therefore, stilbene and benzothiazole derivatives, firstly used to explore amyloid accumulation throughout grey matter in neurodegenerative disease, might be a better marker of demyelination compared to MRI in MS [14].

Bodini and colleagues, used [^11^C]PIB PET to image myelin using dynamic acquisitions that has a short half-life (about 20 min) that limits its clinical viability, whereas Fluorine-18 labelled PET tracers have longer half-life (about 120 min), which allows for a clinical use in patients MS. However, by combining PET and MRI Bodini and colleagues demonstrated that myelin changes, detected with amyloid PET imaging, within lesions was associated with physical disability [15]. Therefore, suggesting a relationship between myelin disruption and outcome.

In this study we adopted a hybrid PET-MR approach using [^18^F]florbetapir PET to quantify myelin content in MS patients. We investigated the relationship between [^18^F]florbetapir uptake and MRI measures, e.g. diffusion tensor imaging and arterial spin labelling together with standard measures of cortical grey matter volume. Finally, we compared SUV_70–90_ analyses with the DVR kinetic modelling to estimate the clinical applicability of PET imaging through simplified semi-quantitative PET measures.

## Materials and methods

### Subjects

Eighteen patients with a definite diagnosis of relapsing-remitting MS according to the 2010 revised McDonalds criteria [16] and twelve healthy controls were recruited in the study at King’s College Hospital NHS Foundation Trust and through advertisement. All participants were successfully screened according to scanning safety criteria (http://www.mrisafety.com; https://www.gov.uk/government/publications/arsac-notes-for-guidance) and had no history of other neurological or psychiatric disorders. All procedures performed in studies involving human participants were conducted in accordance with the principles of the Declaration of Helsinki and national regulations. The study was approved by the local Ethics Committee (London-South East Research Ethics Committee).

All subjects underwent a battery of clinical assessments, including the assessment of clinical disability through the expanded disability status scale (EDSS), the Timed 25-ft walk and the 9-Hole-Peg test. We also assessed the disease severity through the MS severity score (MSSS) before PET-MRI scan. One MS patient dropped out because he was unable to tolerate PET/MR scan due to trigeminal neuralgia. Demographic, clinical and imaging characteristics of the MS patients and healthy controls are summarized in Table 1.

**Table 1:** Demographic, clinical and radiological features of multiple sclerosis patients and healthy controls

	Multiple sclerosis patients	Healthy controls	*P*
Number of subjects	12	18	–
Gender (male/female)	6/12	5/8	0.768
Age, mean ± SD (years)	43.89 ± 8	39.31 ± 9.17	0.150
Disease duration, mean ± SD (years)	10.97 ± 6.90	–	–
Annualized relapse rate, mean ± SD	0.73 ± 0.41	–	–
Disease modifying therapy	No Treatment, N (%): 4 (22.2)	–	–
	Teriflunomide, N (%): 1 (5.6)	–	–
	Dimethyl fumarate, N (%): 5 (27.8)	–	–
	Fingolimod, N (%): 4 (22.2)	–	–
	Daclizumab, N (%): 1 (5.6)	–	–
	Natalizumab, N (%): 1 (5.6)	–	–
	Alemtuzumab, N (%): 2 (11.1)	–	–
Expandend disability status scale, mean ± SD	3.33 ± 1.25	–	–
Multiple sclerosis severity score, mean ± SD	4.53 ± 1.77	–	–
Timed 25-Foot walk, mean ± SD (sec.)	11.06 ± 4.45	6.23 ± 0.93	0.115
Nine-hole pegboard test, mean ± SD (sec.)	24.37 ± 5.60	18.83 ± 1.90	0.009
Normalized cortical grey matter, mean ± SD (cm^3^)	631 ± 54	641 ± 52	0.617
Normalized normal appearing white matter, mean ± SD (cm^3^)	735 ± 29	777 ± 34	0.001
T2 lesion load, mean ± SD (cm^3^)	4.92 ± 5.19	–	–
‘Black hole’ lesion load, mean ± SD (cm^3^)	2.12 ± 2.16	–	–

SD = Standard Deviation

*Only one patient presented a single gadolinium-enhancing lesion

### PET-MRI acquisition

PET images were acquired using a 3-Tesla Magnetom Biograph mMR PET/MR hybrid scanner (Siemens, Erlangen, Germany) with a spatial resolution of 4.3 mm and a FOV of 258 mm at St Thomas PET centre. After the intravenous bolus injection of approximately 300 MBq of [^18^F]florbetapir, we performed a dynamic 90-min scan. PET dynamic images included 27 frames of data (8 × 15, 3 × 60, 5 × 120, 5 × 300 and 5 × 600 seconds). MRI sequences included pre- and post-gadolinium three-dimensional magnetization-prepared rapid gradient-echo T1 (three-D-T1w MPRAGE, TR [repetition time]: 1700 msec, TE [echo time]: 2.63 msec, TI [inversion time]: 900 msec, flip angle: 9°, pixel size: 1.1 × 1.1 × 1.1 mm), fast fluid-attenuated inversion recovery (FLAIR, TR: 5000 msec, TE: 499 msec, TI: 1800 msec, pixel size: 1x1x1mm) and turbo spin-echo T2-weighed images (T2w TSE, TR: 3200 msec, TE: 409 msec, TI: 900 msec, pixel size: 1x1x1mm). For each subject a diffusion tensor echo-planar imaging sequence was registered, with generalized auto-calibrating partial parallel acquisition, at 2-fold acceleration (TR: 9300 msec, TE: 88 msec, pixel size: 1.9 × 1.9x2mm, acquisition matrix: 128 × 128, field of view: 240 mm, n° of slice: 40; diffusion-sensitizing pulsed magnetic field gradients were applied in 64 non-collinear directions, at two diffusion weightings; one at 1000s/mm2 and the second at 0 s/mm2) and a single-shot gradient echo-planar imaging sequence was used to assess cerebral blood perfusion through pseudo-continuous arterial spin labelling (TR: 3750 msec, TE: 21.96 msec, pixel size: 4x4x4mm, field of view: 256 mm, flip angle: 180°, number of slices: 26, label duration: 1500, post label delay: 1800 ms, 30 tag control pair).

## Image data analysis

### [^18^F]florbetapir PET data

Individual PET frames were realigned to a common reference frame with high signal-to-noise ratio, to perform an intra-subject motion correction [17]. Fluorine-18 decay correction was applied. We performed a reference region approach through a supervised cluster method to avoid blood sampling, which is a cumbersome and bothersome procedure for patients.

The supervised clustering algorithm for [^18^F]florbetapir was implemented following procedures already applied to [^11^C]PIB [18]. Specifically, each frame’s radioactivity was normalized for every voxel in the brain by subtracting the mean frame activity and dividing it by the frame standard deviation. Three kinetic classes for [^18^F]florbetapir were defined for the normalized PET dynamic sequence using the twelve healthy controls, who received [^18^F]florbetapir PET in this study: normal appearing grey matter (class 1), blood pool (class 2) and white matter (class 3). Class 1 and 3 were calculated from the average of normalized time activity curve for [^18^F]florbetapir in the grey matter and white matter mask respectively. Class 2 was defined by extracting the 30 voxels with the highest [^18^F]florbetapir radioactivity concentration over the first 60 s of PET acquisition, corresponding to the big blood vessels. The supervised cluster algorithm performs multiple linear regressions for each voxel in the grey matter in order to model its kinetic as a linear combination of the predefined kinetic classes for [^18^F]florbetapir. For each voxel the weight from each class was calculated. Voxels with a class 1 weighted ratio higher than 0.9 were selected as reference regions and the time activity curve in this area was calculated as the average of the non-normalized voxel time activity curve.

Logan graphical reference method was applied at a voxel level on PET scans in native space to produce a parametric maps of [^18^F]florbetapir binding measured as the DVR [19].

[^18^F]florbetapir PET was also quantified using standardized uptake value (SUV) in the 70–90 min after injection. SUV_70–90_ map was generated by correcting absolute radioactivity concentrations (C; kBq/ml) for subject body weight (BW; kg) and injected dose (ID; MBq): SUV=C/(ID/BW). [^18^F]florbetapir SUV_70–90_ in the cerebellar cortex was compared between MS patients and healthy controls; if no differences were detected, the SUVR map would be calculated.

### Conventional MR images post-processing

The following regions of interest have been defined by two experienced observers (A.C., Z.C.) on each subject’s image, using Analyze medical imaging software (version 12, Mayo Foundation AnalyzeDirect): (a) T1 lesions, defined as hypointense areas in 3D-T1w MPRAGE compared to normal appearing white matter (NAWM); (b) T2 lesions, defined as hyperintense areas in FLAIR compared to NAWM; (c) Gadolinium enhancing (Gd+) lesions; (d) inner in-plane 2D perilesional layer outside T2 lesions (4 to 0 mm from the lesion); (e) Outer in-plane 2D perilesional layer outside T2 lesions (8 to 4 mm from the lesion). Each T2 Lesion was also divided in two infralesional layers. A third experienced operator (G.D.), blind to clinical information, solved discordances between the two observers.

T2 lesions and T1 lesions were further classified according to regional location (infratentorial, periventricular, white matter lesions in the corona radiata, defined as white matter lesions to differentiate among other lesion location, and juxtacortical) by two experienced observers (A.C., Z.C.). To reduce potential bias from partial volume effects lesions smaller than the PET resolution were not considered and perilesional layers larger than the PET resolution were excluded.

T1 lesions mask was used to perform a ‘lesion filling’ procedure [20] on the 3D-T1w MPRAGE. We used T1 filled images for MS patients and T1 images for healthy controls to segment brain tissues in white matter, grey matter and CSF through the SPM12 software (Statistical Parametric Mapping version 12; http://www.fil.ion.ucl.ac.uk/spm/software/spm12/) with a probability of belonging to each tissue class higher than 90%. Cortical grey matter was calculated by subtracting the deep grey matter mask, obtained through the FIRST tool available in FMRIB Software Library (FSL; University of Oxford, UK) [21], from the total grey matter mask. In order to extract the cerebellum cortex as reference region for the SUVR, grey matter was automatically segmented using the multi-atlas propagation with enhanced registration (MAPER) approach [22]. After visual inspection for multi-atlas propagation with enhanced registration segmentation errors, the cerebellum grey mater cortex was extracted. Cortical grey matter volume and white matter volume, normalized for subject head size, were estimated using SIENAX [23].

### Advanced MR images post-processing

Gradient vector (bvec) and b-value (bval) files were produced and merged to b matrix files to process DTI images. Composition of mean diffusivity, fractional anisotropy, radial diffusivity and axial diffusivity maps were undertaken using tools from the FMRIB Software Library (FSL, https://fsl.fmrib.ox.ac.uk/fsl/). FMRIB’s [24]. Top-up and eddy, motion-susceptibility and eddy-current fields were generated to correct for off-field resonance and movement-related distortions [25, 26]. Voxel-by-voxel diffusion tensors were applied to the diffusion-weighted image through DTIfit, generating the quantitative mean diffusivity, fractional anisotropy, radial diffusivity and axial diffusivity maps. These maps were linearly registered into the corresponding DVR parametric map through FMRIB’s Linear Image Registration Tool (FLIRT) [27].

CBF map was calculated following Alsop and colleagues approach [28]. Once label and control images were corrected for motion through MCFLIRT [27], CBF parametric map was produced. CBF is measured in ml/100 g/min.

### PET-MRI co-registration

MRI scans and DTI maps were linearly registered into the corresponding DVR parametric maps through FLIRT. The derived transformation matrix was applied to align each region of interest to subjects’ DVR parametric map. T2 and T1 lesions maps in DVR space were summed and the combined mask was subtracted from the total white matter to obtain the NAWM mask. In order to evaluate the CBF in damaged white matter areas for healthy controls, we defined a DVR cut-off for myelin integrity as the mean DVR in the white matter minus 1.96 times the standard deviation, corresponding to the 5th percentile. We selected for each patients’ DVR parametric map, voxels belonging to white matter with a DVR value lower than the cut-off creating a binary mask. The resulting mask corresponded to the myelin impaired white matter regions and the remaining voxels were classified as unaffected white matter. Both impaired and unaffected white matter masks were linearly registered into the corresponding CBF parametric map through FLIRT and the cerebral perfusion was assessed. All analyses were performed in DVR space to avoid partial volume effect for DVR map.

### Statistical analysis

Statistical analyses were performed using Stata software (version 13; StataCorp LP, College Station, TX). Variance homogeneity and Gaussianity were tested for each variable with Shapiro-Wilk test.

Differences in white and grey matter [^18^F]florbetapir DVR between MS patients and healthy controls were tested using a general linear model including the group as factor of interest, age and gender as covariates and DVR as the dependent variable. Differences in [^18^F]florbetapir DVR between MS patients’ normal appearing, healthy controls’ white matter, and each defined region of interest were assessed using a mixed-effect linear model including regions of interest as the factor of interest, age and gender as covariates, the subject as the random effect and the DVR as the dependent variable. The relationship between lesion intensity on conventional T1 and T2 MRI images and DTI measures and [^18^F]florbetapir DVR values was further explored using a mixed-effect linear model including each lesion’s intensity or DTI measures as the factor of interest, age and gender as covariates, the subject as the random effect, and each lesion’s DVR as the dependent variable. The potential additional value of DVR over fractional anisotropy as measured through DTI in differentiating normal appearing white matter, outer T2 perilesion layer and inner T2 lesion layer was investigated via hierarchical linear regression analyses. In particular, age, gender and fractional anisotropy were entered in a first step using the brain region (normal appearing white matter, outer T2 perilesion layer and inner T2 perilesion layer) as dependent variable. We chose the fractional anisotropy measure, as it is the most widely used measure from DTI to assess microstructural changes in white matter. The second step added DVR in order to evaluate the additional value of such measure in distinguish between the aforementioned brain regions. The association between [^18^F]florbetapir DVR and cortical and NAWM normalized volume was assessed through a mixed-effect linear model including mean T1 and T2 lesions DVR as the factor of interest, age and gender as covariates, the subject as the random effect, and the cortical and NAWM normalized volume as the dependent variable.

The cerebral blood flow in NAWM was compared to intact and damaged white matter tissue using a mixed-effect linear model using regions of interest (MS patients’ NAWM, healthy controls’ white matter, impaired white matter, unaffected white matter) as factors of interest, and the cerebral blood flow as the dependent variable to explore how cerebral blood flow changes in brain regions with damaged myelin.

The diagnostic accuracy for both SUV and DVR in classifying white matter tissue as belonging to NAWM or demyelinated tissue (namely, the manually definedT2 lesions) was estimated through a receiver operating characteristic analysis for DVR and SUV_70–90_ and a comparison between the two areas under the curve was performed. We tested the difference between the SUV in the cerebellum cortex for MS patients and healthy controls through an unpaired two-tailed Student-t test. We also explored the correlation between DVR and SUV_70–90_ in NAWM, T2 and T1 lesion through a linear regression including T2 and T1 lesions mean SUV as the factor of interest, age and gender as covariates DVR as the dependent variable.

The correlation between DVR in both T2 lesions and black holes and physical disability assessed through the expanded disability status scale, the timed 25-ft walk and the 9-Hole-Peg test, and disease severity assessed through the MS severity score, was evaluated through a linear regression including T2 and T1 lesions mean DVR as the factor of interest, age and gender as covariates and the clinical outcome as the dependent variable. Data will be provided upon request.

All data are presented as mean ± standard deviation (SD), and the specificity level *α* was set for all comparisons at *P* < 0.05.

## Results

### [^18^F]florbetapir PET DVR

**Fig. 1:**
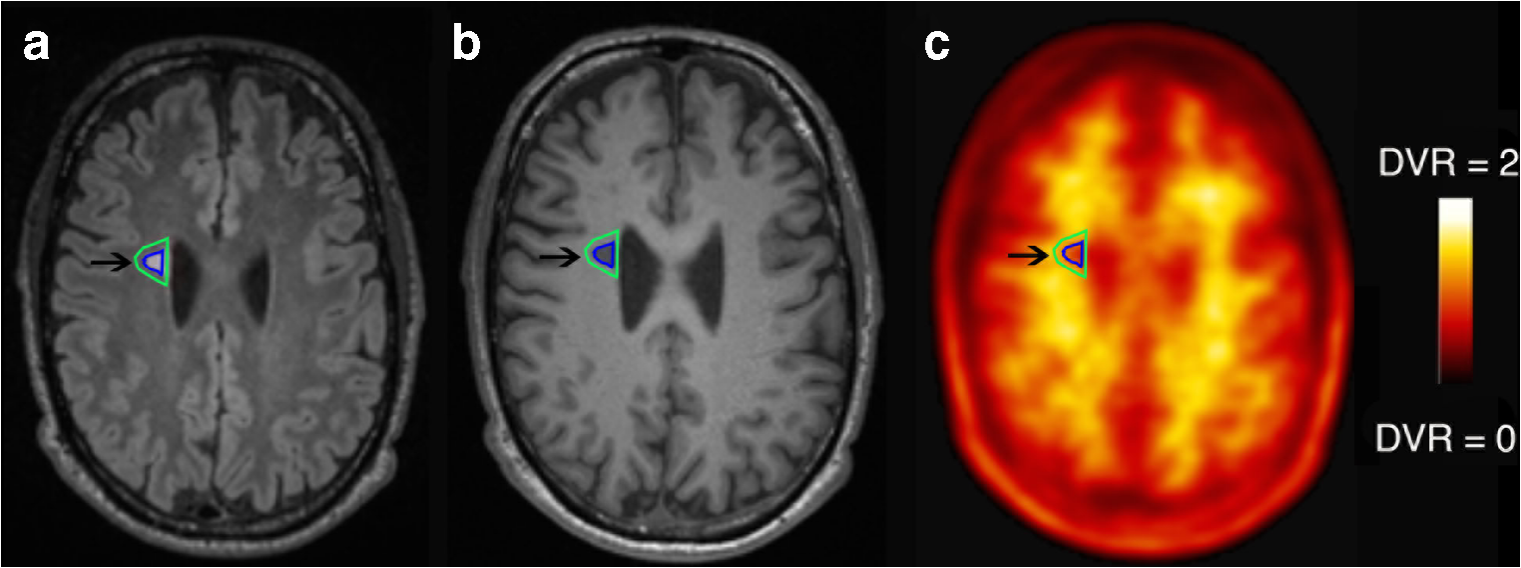
[^18^F]Florbetapir PET and MRI images from a Multiple Sclerosis patient. 3D-T1w MPRAGE image (a), FLAIR image (b) [^18^F]florbetapir distribution volume ratio **(**DVR) parametric map (c) for a 54-year-old female relapsing-remitting Multiple Sclerosis patient (disease duration = 10 years; Annualized-Relapse-Rate of 0.3; EDSS = 3.5; and MSSS = 4.55). Arrows point to T1 lesions (a) and T2 lesions (b) showing a reduced [^18^F]florbetapir binding in the DVR parametric map (c). Colour bar reflects range of [^18^F]florbetapir DVR. Arrows point to demyelinating lesion, with both the lesion (in blue) and perilesional rim (in green). In perilesional area (up to 8 mm) DVR values were reduced compared with the white matter as defined through standard MRI in healthy controls (estimated 7% loss, *P* < 0.01)

**Fig. 2:**
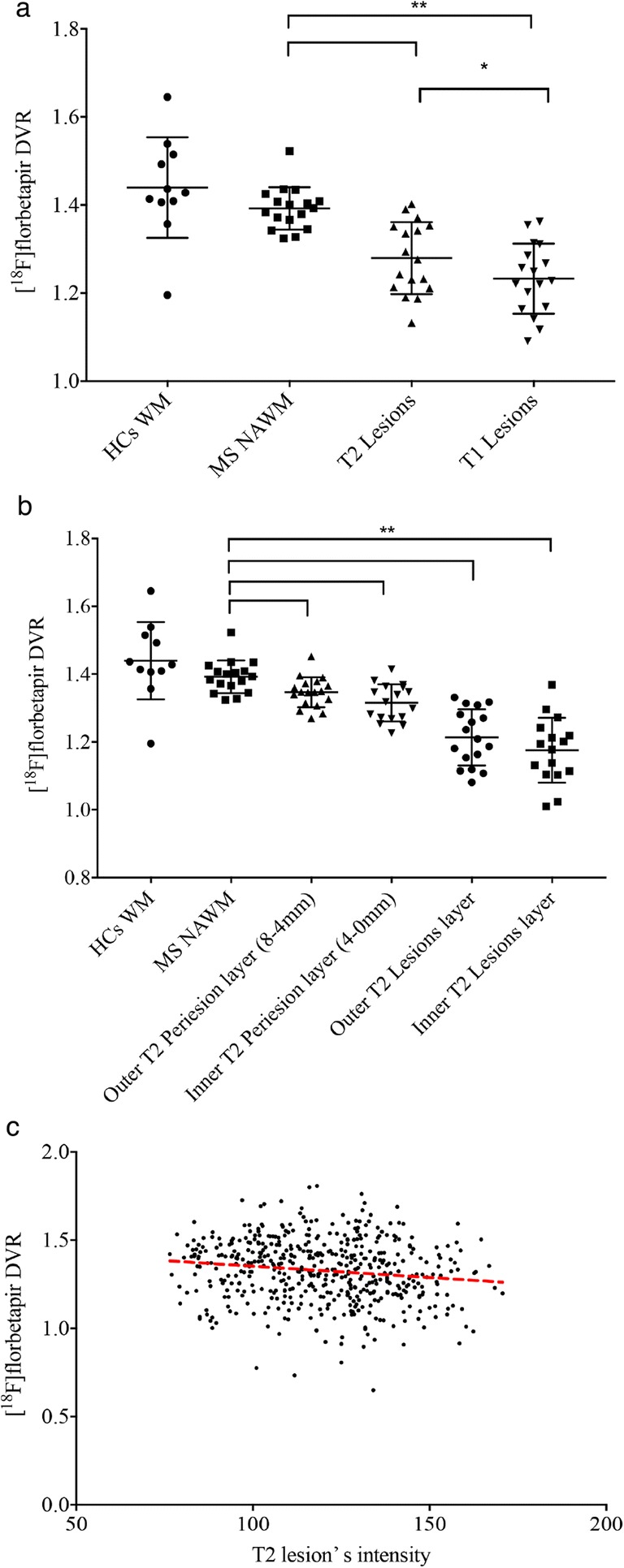
Relationship between [^18^F]florbetapir PET and T1 and T2 white matter lesions. (a) Scatter plot diagram showing mean [^18^F]florbetapir distribution volume ratio **(**DVR) and standard deviation in white matter for healthy controls and in normal appearing white matter, T2 lesions and T1 lesions in Multiple Sclerosis patients. Mean DVR decreased from normal appearing white matter to T2 lesions and the lowest value was found in T1 lesions. ** *P* < 0.001, paired two-tailed student’s t test. (b) Scatter plot diagram shows mean DVR and standard deviation in white matter for healthy controls and in normal appearing white matter, outer T2 perilesional layer (8–4 mm), inner T2 perilesional layer (4–0 mm), outer T2 intralesional layer, middle T2 intralesional layer and inner T2 intralesional layer for Multiple Sclerosis patients. Mean DVR values decreased from normal appearing white matter to the center of T2 lesions. ** P < 0.001, paired two-tailed student’s t test. (c) Scatter plot diagram showing the correlation between mean DVR and T2 lesions intensity. Coefficient and *P* value were calculated using a mixed-effect linear model including each lesion’s intensity as factor of interest, age and gender as covariates, subject identification number as random effect and each lesion’s DVR as dependent variable. The scatter plot displays a direct correlation between T2 lesion intensity and mean DVR values (coeff. = − 0.002, P < 0.001). Lower DVR values were found in brighter lesions

Both T2 and T1 lesions showed a lower mean DVR in MS patients compared to healthy controls’ white matter (−11.1%, *P* < 0.001, −14.6%, *P* < 0.001 respectively; Table 2 and 3, Fig. 1 and 2a).

**Table 2:** Mean [^18^F]florbetapir DVR for each region of interest in multiple sclerosis patients and healthy controls

Region of Interest	Multiple Sclerosis patients	Healthy controls
Cortical grey matter, mean DVR ± SD (Min - Max)	1.02 ± 0.06 (0.95–1.17)	1.05 ± 0.09 (0.84–1.20)
Normal appearing white matter, mean DVR ± SD (Min - Max)	1.39 ± 0.05 (1.32–1.52)	1.44 ± 0.11 (1.19–1.65)
T2 lesions, mean DVR ± SD (Min - Max)	1.28 ± 0.08 (1.13–1.40)	–
T1 lesions, mean DVR ± SD (Min - Max)	1.23 ± 0.08 (1.09–1.36)	–
T2 mild intensity lesions, mean DVR ± SD (Min - Max)	1.23 ± 0.08 (1.10–1.36)	–
T2 moderate intensity lesions, mean DVR ± SD (Min - Max)	1.19 ± 0.08 (1.06–1.33)	–
T2 severe intensity lesions, mean DVR ± SD (Min - Max)	1.19 ± 0.14 (0.99–1.42)	–
T1 mild intensity lesions, mean DVR ± SD (Min - Max)	1.37 ± 0.09 (1.23–1.61)	–
T1 moderate intensity lesions, mean DVR ± SD (Min - Max)	1.26 ± 0.08 (1.12–1.40)	–
T1 severe intensity lesions, mean DVR ± SD (Min - Max)	1.11 ± 0.12 (0.83–1.35)	–
Outer T2 perilesion layer (8–4 mm), mean DVR ± SD (Min - Max)	1.35 ± 0.05 (1.27–1.45)	–
Inner T2 perilesion layer (4–0 mm), mean DVR ± SD (Min - Max)	1.32 ± 0.05 (1.22–1.41)	–
Outer T2 intralesional layer, mean DVR ± SD (Min - Max)	1.21 ± 0.08 (1.08–1.33)	–
Inner T2 intralesional layer, mean DVR ± SD (Min - Max)	1.17 ± 0.10 (1.01–1.37)	–
Infratentorial, mean DVR ± SD (Min - Max)	1.37 ± 0.13 (1.13–1.55)	–
Periventricular, mean DVR ± SD (Min - Max)	1.17 ± 0.09 (1.02–1.35)	–
Juxtacortical, mean DVR ± SD (Min - Max)	1.18 ± 0.15 (0.85–1.44)	–
White matter lesions, mean DVR ± SD (Min - Max)	1.36 ± 0.06 (1.27–1.49)	–

DVR = Distribution Volume Ratio; SD = Standard Deviation

*Only one patient presented a single gadolinium-enhancing lesion

**Table 3:** Differences in mean [^18^F]florbetapir DVR between regions of interest in multiple sclerosis patients and mean DVR in white matter for healthy controls

Independent variables	Coefficient β	SE	95% CI	*z*	*P*
Normal appearing white matter	- 0.04	0.03	- 0.10 0.02	- 1.38	0.17
T2 lesions	- 0.15	0.03	- 0.21 - 0.10	- 5.15	<0.001
T1 lesions	- 0.20	0.03	- 0.26 -0.14	- 6.71	<0.001
T2 mild intensity lesions	- 0.11	0.04	- 0.18 - 0.04	- 3.00	0.003
T2 moderate intensity lesions	- 0.23	0.04	- 0.28 - 0.10	- 6.44	<0.001
T2 Severe intensity lesions	- 0.24	0.04	- 0.31 - 0.16	- 6.49	<0.001
T1 mild intensity lesions	- 0.07	0.03	- 0.17 0.00	- 1.88	0.06
T1 moderate intensity lesions	- 0.18	0.03	- 0.25 - 0.11	- 4.97	<0.001
T1 Severe intensity lesions	- 0.32	0.03	- 0.39 - 0.26	- 9.30	<0.001
Outer T2 perilesional layer (8–4 mm)	- 0.09	0.03	- 0.15 - 0.03	- 2.86	0.004
Inner T2 perilesional layer (4–0 mm)	- 0.12	0.03	- 0.18 - 0.06	- 3.89	<0.001
Outer T2 intralesional layer	- 0.22	0.03	- 0.28 - 0.16	- 7.24	<0.001
Inner T2 intralesional layer	- 0.25	0.03	- 0.31 - 0.19	- 8.34	<0.001
Infratentorial	- 0.07	0.04	- 0.15 0.01	- 1.79	0.07
Periventricular	- 0.28	0.04	- 0.35 - 0.20	- 7.01	<0.001
Juxtacortical	- 0.26	0.04	- 0.34 - 0.18	- 6.58	<0.001
White matter lesions	- 0.08	0.04	- 0.16 - 0.01	- 2.09	0.04

Values are adjusted for age and gender. SE = Standard Error, CI = Confidence Interval; DVR = Distribution Volume Ratio

None of the enrolled MS patients showed Gd + lesions. No differences were found between mean DVR in cortical grey matter for MS patients and healthy controls (*coeff.* = −0.02, *P* = 0.50, confidence interval [CI] = −0.7 to 0.04) and between NAWM in MS patients and white matter DVR in healthy controls (*coeff.* = −0.05, *P* = 0.12, CI = −0.11 to 0.01).

Compared to healthy controls mean DVR in white matter, lower mean DVR values were found in the outer T2 perilesional layer (8–4 mm) (−6.3%; *P* = 0.004), in the inner T2 perilesional layer (4–0 mm) (−8.3%; *P* < 0.001), in the outer T2 intralesional layer (−16%; P < 0.001) and in the inner T2 intralesional layer (−18.8%; *P* < 0.001) (Tables 2 and 3; Fig. 2b). At lesion-based analysis, T2 lesions intensity was inversely correlated to the mean DVR after correction for age and gender (*r* = −0.002, *P* < 0.001; Fig. 2c).

Similarly, T1 lesions with a lower intensity showed a lower mean DVR after correction for age and gender (*coeff.* = 0.003, *P* < 0.001).

Using regional classification, mean DVR in infratentorial lesions was not different compared to mean DVR in white matter for healthy controls. Conversely, a lower mean DVR was found in periventricular (−18.8%; *P* < 0.001), juxtacortical (−18.1%; *P* < 0.001) and white matter T2 lesions (−5.6%, *P* = 0.04) compared to healthy controls’ white matter mean DVR (Tables 2 and 3).

Mean T2 and T1 lesions DVR was not related to the annualized relapse rate, the disease duration, the EDSS, the 9-Hole-peg test and the MSSS after correction for age and gender. Higher DVR within T2 lesions was associated with lower Timed 25-ft walk time after correction for age and gender (*coeff.* = − 9.42, *P* < 0.001).

### Correlation of [^18^F]florbetapir PET and MRI measures

MS patients had a lower normalized NAWM volume compared to healthy controls’ white matter (*P* = 0.001). No differences were found for cortical grey matter. Normalized cortical grey matter and normalized white matter volume were not associated with the mean DVR in T2 and T1 lesions after correction for age and gender.

**Fig. 3:**
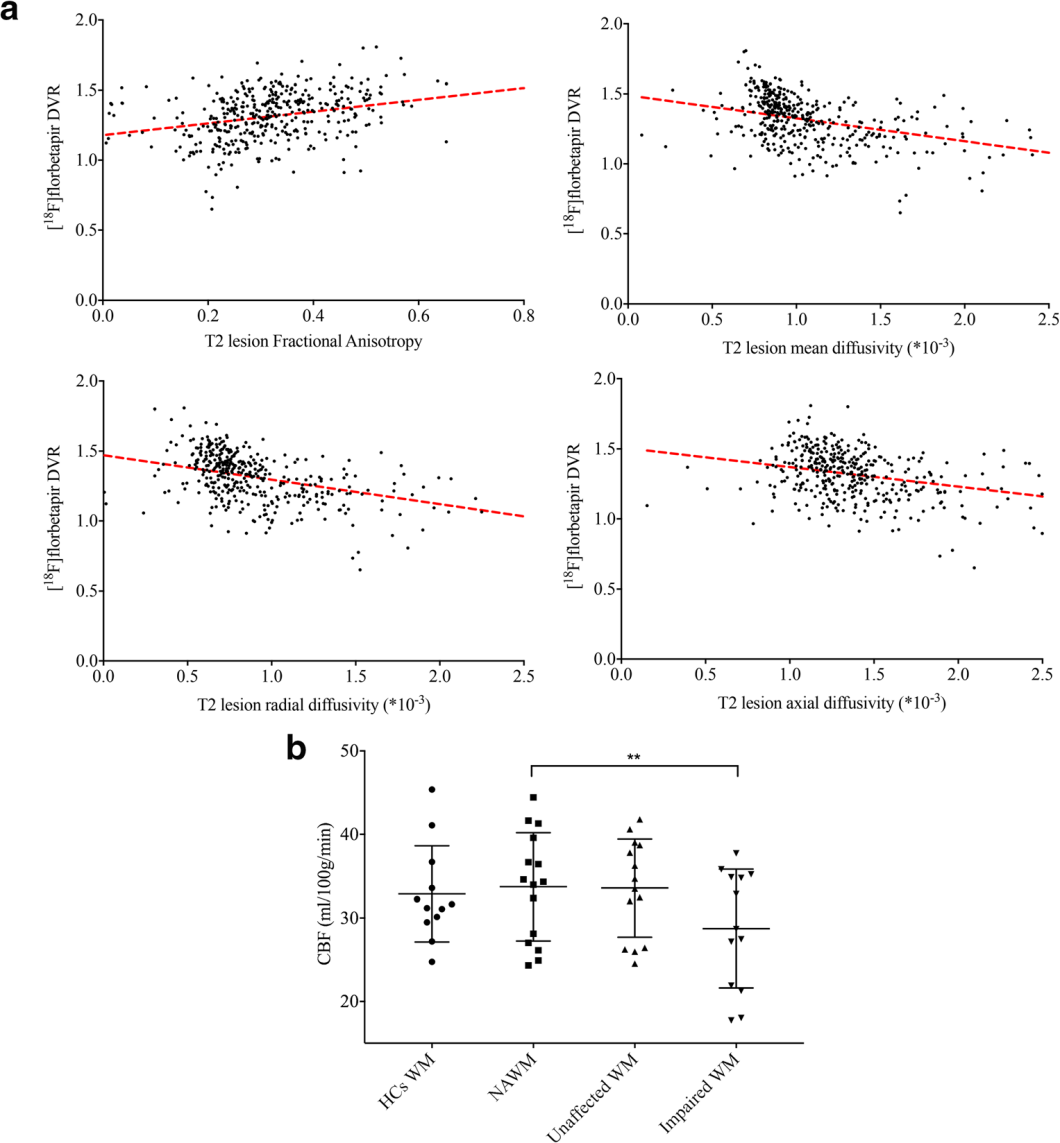
Relationship between [^18^F]florbetapir PET and advanced MRI measures. (a) Scatter plot diagram displays the relationship between mean [^18^F]florbetapir distribution volume ratio **(**DVR) and fractional anisotropy, mean, axial and radial diffusivity in T2 lesions. DVR decreased significantly in lesions with lower fractional anisotropy (coeff. = 0.47, P < 0.001) and increased in lesions with higher mean diffusivity (coeff. = −0.148, P < 0.001), axial diffusivity (coeff. = −0.123, P < 0.001) and radial diffusivity (coeff. = −0.163, P < 0.001). (b) Scatter plot diagram shows mean cerebral blood perfusion and standard deviation in white matter for healthy controls and in normal appearing white matter, unaffected white matter and impaired white matter for Multiple Sclerosis patients. Impaired white matter areas showed a significantly lower perfusion. ** P < 0.001, paired two-tailed student’s t test

Using a lesion-based approach, mean DVR in T2 and T1 lesions correlated with fractional anisotropy (*coeff.* = 0.47 and coeff. = 0.50, respectively; *P* < 0.001),mean diffusivity (*coeff.* = −0.148 and *coeff.* = −0.238, *respectively; P* < 0.001), axial diffusivity (*coeff.* = −0.123 and *coeff.* = −0.176, respectively; P < 0.001) and radial diffusivity (*coeff.* = −0.163 and *coeff.* = −0.227, respectively; *P* < 0.001) (Fig. 3a). DVR resulted to be more sensitive to brain damage when compared with fractional anisotropy. Specifically, the model for predicting brain damaged areas (normal appearing white matter, outer T2 perilesion layer and inner T2 perilesion layer) including DVR (R^2^ = 0.39, F = 1.94, with *coeff.* For DVR = -1.81, CI = −3.59 to −0.02, namely the inner the T2 lesion we go the lower the DVR value) was more sensitive than the model only including fractional anisotropy (R^2^ = 0.04, F = 0.19) (*P* = 0.04).

Mean CBF was not different between unaffected MS patients’ white matter areas and healthy controls’ white matter (31.31 ± 6.53 and 33.73 ± 6.50, *P* > 0.05). Conversely, mean CBF was significantly reduced in impaired white matter tissue compared to NAWM in MS patients (% reduction in mean CBF was 10.9%, *P* = 0.005; Fig. 3b).

### Comparison between [^18^F]florbetapir SUV, SUVR and DVR

**Fig. 4:**
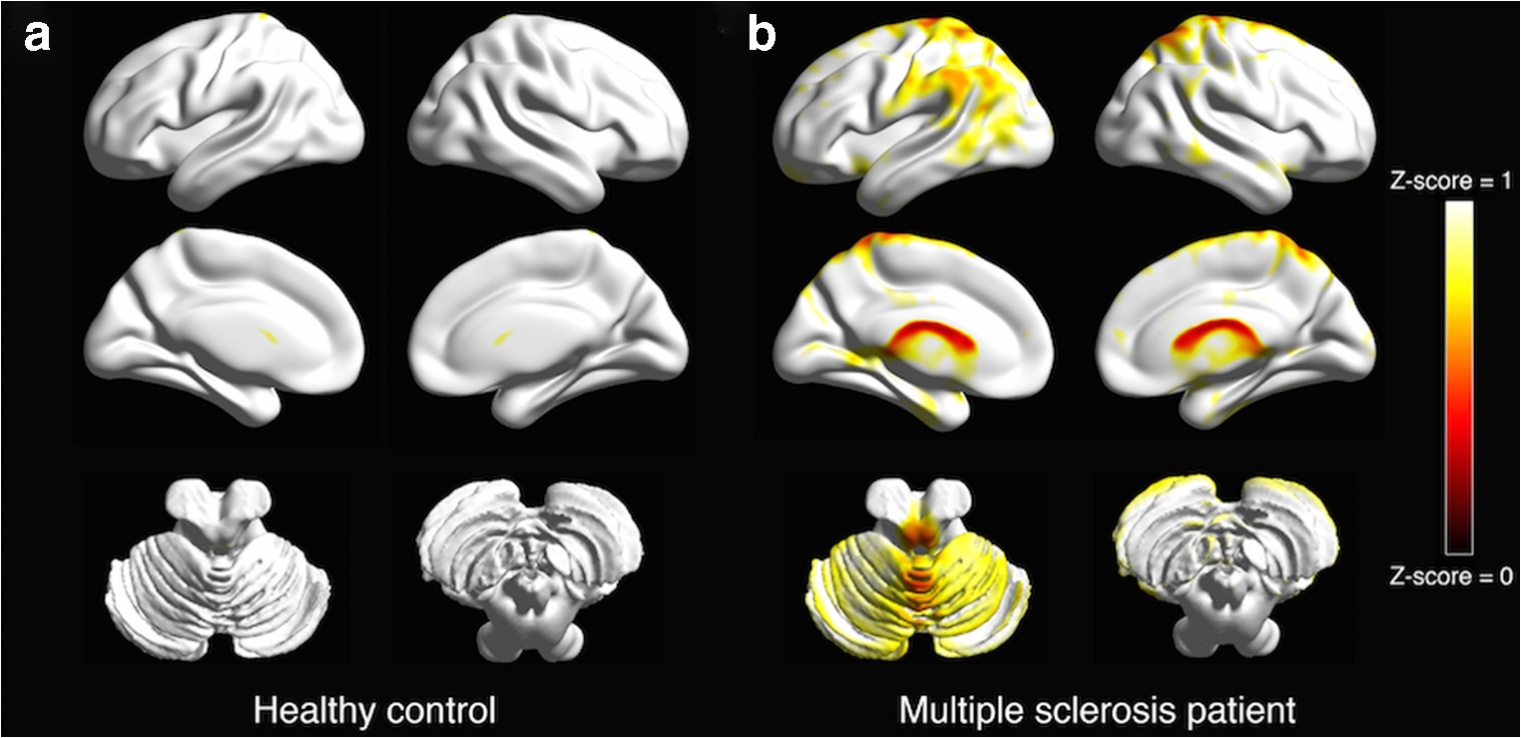
Mean [^18^F]florbetapir SUV_70–90_ in cortical grey matter. Cortical regions showing a mean SUV_70–90_ lower than the calculated cut-off for normal cortical myelination. (a) Healthy 38-year-old female and (b) 44-year-old female relapsing-remitting Multiple Sclerosis patient (disease duration = 18 years; Annualized-Relapse-Rate of 0.22; EDSS = 1; MSSS = 0.26). Cut-off was calculated as the mean of SUV_70–90_ in the cortical grey matter for healthy controls – 1.96 times standard deviation, which corresponds to the 5th percentile. Calculated cut-off was 1.0246. Images presented on the International Consortium for Brain Mapping (ICBM) whole brain surface visualized in BrainNet Viewer (http://www.nitrc.org/projects/bnv/); colour bar represents Z-scores

All the previous analyses were performed using the SUV_70–90_ as dependent variables instead of mean DVR. We found similar results to those obtained using mean DVR (data not shown). However, when evaluating the SUV_70–90_ in the cerebellar cortex for MS patients and healthy controls to perform the SUVR analysis, we found that SUV_70–90_ was lower in the cerebellar grey matter for MS patients compared to healthy controls (*P* = 0.04; Fig. 4). Therefore, we did not perform the analysis using the SUVR as measure of tracer binding. The comparison between the receiving-operator curves for DVR and SUV in distinguish between NAWM and demyelinated lesions as detected through T2 MRI sequence revealed no difference (area under the curve 0.77 and 0.66, respectively; *P* value for ROC curves comparison = 0.26). Moreover, DVR correlated with SUV in NAWM (*coeff.* = 0.06, *P* = 0.03), T2 and T1 lesions (*coeff.* = 1.70 and *coeff.* = 0.21, respectively; *P* < 0.001).

## Discussion

In this study, we demonstrated that [^18^F]florbetapir PET is a valid tool to detect the pattern of demyelination in MS, with a comparable accuracy between SUV_70–90_ PET measure and the computational DVR analysis methods. Combining PET with advanced MRI techniques, we showed that myelin damage was correlated with microstructural white matter changes and lower perfusion rate, providing additional information.

We showed that [^18^F]florbetapir binding from dynamic DVR and static SUV measures have comparable accuracy in differentiating myelin damaged tissue from NAWM. [^18^F]florbetapir quantification with SUV has a number of advantages including short scan-time, with acquisition within a predefined time window and lower costs compared to the dynamic continuous acquisition. Bodini and colleagues demonstrated that longitudinal [^11^C]PIB PET imaging scans quantified through the DVR, might depict not only demyelination but also remyelination over time [15]. Hence SUV measures could be helpful when assessing treatment efficacy in clinical trials and in the management of patients with MS. However, the longitudinal reproducibility of Logan DVR and SUV for [^18^F]florbetapir PET might deserve further studies to be confirmed.

We showed that [^18^F]florbetapir binding was decreased in focal white matter lesions of the MS patients, defined on conventional MRI, compared to the tracer uptake in the white matter of the healthy controls. T1 lesions showed the lowest value, confirming that they are characterized by extensive myelin destruction [29]. Post-mortem studies have already reported that T1 hypointensity reflects histopathological modifications, such as axonal loss, extracellular oedema and demyelination [30]. The extent of each of these pathological processes could not be evaluated through conventional MRI. To overcome this obstacle, several advanced MRI techniques have been applied in MS. Among them, the diffusion tensor imaging is the most promising technique to assess microstructural changes while measuring the water motility within tissues. However, the diffusion tensor imaging measures the global structural modifications within damaged tissues including demyelination, axonal damage, microglia activation [31] and gliosis without being able to differentiate them [32, 33]. [^18^F]florbetapir PET imaging can measure the extent of demyelination without including biases from other pathological changes, such as gliosis or axonal loss, and we demonstrated that DVR provides additional information on tissue damage compared to DTI measures. Further studies including other advanced MRI techniques such as magnetization transfer imaging might also evaluate the specificity of PET imaging over MRI in detecting demyelination.

[^18^F]florbetapir binding was similar for MS patients and healthy controls in NAWM. The absence of difference in myelin content of grey and white matter that we found for the two groups appears not to be in line with previous studies that have shown tissue changes within both grey and white matter outside the lesions detected by conventional MRI through diffusion tensor imaging, magnetisation transfer imaging and spectroscopy [32, 34–36]. However, this discrepancy further underlines that such MRI techniques are able to detect global pathological changes, including axonal degeneration, gliosis and inflammation throughout the brain, but are not specific measures of myelin integrity. Noteworthy, it is also important to underline that demyelination arises to different extent from neuroinflammation. Therefore, although microglia activation was reported throughout the normal appearing white matter [37], demyelination might not occur simultaneously. However, we reported that the white matter tissue surrounding the edge of demyelinating lesions, detected through conventional MRI, is also affected by demyelination at different degrees. A post-mortem study by De Groot and colleagues reported reduced myelin density in areas in close proximity to the demyelinating damaged areas in brain specimens from MS patients [30]. Furthermore, Bodini and colleagues reported a similar pattern of demyelination through a [^11^C]PIB PET imaging study [15]. In this latter study, authors evaluated up to 2 mm rim surrounding T2 lesions. Here, we reported that myelin disruption is a process going further beyond the borders detected through conventional MRI images, up to almost 1 cm. This aspect is important when assessing the extent of demyelination throughout the CNS. Slowly evolving lesions are becoming a hallmark for disease progression in MS [38]. Noticeably, our finding suggested that [^18^F]florbetapir PET imaging might detect with a single scan a larger areas of damaged tissue compared to conventional MRI and, thus, might be considered a better predictor of disease progression. Moreover, the increased size of a lesion may also depend on an ineffective and incomplete process of remyelination and does not necessarily implies the reactivation of the lesion. Interestingly, longitudinal [^18^F]florbetapir PET scans might shed further light in the biological bases of the remyelinating process around the lesions.

We would expect a difference in cortical grey matter [^18^F]florbetapir binding between MS patients and healthy controls, because cortical demyelinating lesions are becoming a hallmark in MS pathology [36]. According to this hypothesis, we found that the myelin content within the cerebellar cortex for MS patients was lower than the one in the cerebellar cortex of healthy controls. This finding also highlight that the cerebellum grey matter cortex must not be chosen as reference region for amyloid-β PET imaging in MS as opposed to other neurodegenerative disorders, such as Alzheimer’s disease [39, 40]. This caveat also confirms that the supervised cluster analysis is crucial when selection of the reference region is needed. Moreover, as previously stated, also SUV could be helpful when assessing treatment efficacy; however, its longitudinal reproducibility should to be explored. The extent of demyelination within both T2 and T1 lesions was not associated with cortical or white matter volume. Although several studies have pointed out that the lesion load measured through conventional MRI correlates with the grey matter volume in MS [41, 42], our results suggested that demyelination specifically measured with [^18^F]florbetapir PET imaging within MS lesions per-se is not the cause of brain atrophy. Supposedly, the balance between remyelination, preserved axonal integrity and the wallerian degeneration of axons traversing lesions might impact the total brain volume and the cortical thickness [43]. Moreover, axonal injury in MS is not restricted to white matter lesions but can occur also in the NAWM, independently from focal cerebral demyelination [35]. Therefore, longitudinal amyloid-β PET imaging studies, eventually coupled with [^11^C]flumazenil PET, specifically measuring neurodegeneration [44], are needed to further elucidate the impact of demyelination throughout the CNS on brain and cortical axonal loss. In addition, a combined [^11^C]flumazenil and amyloid-β PET imaging study might also shed further light on the interplay between demyelination, eventually remyelination and axonal degeneration. A recent paper, using a complex approach involving optical coherence tomography, conventional and advanced MRI, reported that demyelination over a specific white matter tract (i. e. optic radiation) precedes neurodegeneration [45]. Using a PET approach using two probes specifically targeting the two main pathological processes occurring in MS patients, might provide the unique opportunity to evaluate the dynamics of such processes with a high degree of specificity and to evaluate the impact of the interplay between these two phenomena on clinical disability.

We observed a reduced cerebral arterial blood perfusion in white matter regions with a higher extent of demyelination. MS lesions and normal appearing white matter are characterized by cerebral hemodynamic impairment even in absence of blood-brain-barrier damage [46], resulting in reduced arterial blood supply. Previous pathological studies have already reported hemodynamic changes in MS including vessels’ thrombosis and tissue hypoxia due to fibrin deposition in demyelinating plaques. Specifically, the high venular density and the reduced arterial flow in the damaged white matter tissue suggest that hemodynamic changes could contribute to tissue damage. Yet, the precise impact of vascular factors on MS pathology has not been fully elucidated. Our data about cerebral blood flow are partly in line with previous findings, especially when compared with studies using the same methodology [47, 48], which will further support the impact of vascular changes on MS pathology. Longitudinal PET/MRI studies could further investigate whether arterial blood flow reduction is the primary cause or the result of the inflammatory-driven tissue necrosis. It is worthy to mention that both DVR and SUV depend on an intact blood-brain barrier and changes in blood-brain barrier integrity occurring in gadolinium enhancing lesions could potentially introduce a bias. However, in the present study only one single gadolinium enhancing lesion was present in one patient and, in addition, DVR is a dynamic measure which take into account the time activity curve of tracer distribution. Furthermore, changes in CBF do not appear to have a significant effect on DVR values estimated using nonlinear modelling or graphical analysis [49]. Therefore, [^18^F]florbetapir DVR measures are unlikely to be significantly influenced by cerebral arterial blood perfusion.

We did not find any correlations between the extent of white matter lesions demyelination and the disease severity, assessed through the MSSS, disease duration and annualized relapse rate and physical disability assessed through the EDSS. However, we found a correlation between physical disability assessed through the Timed 25-ft walk and the extent of myelin loss within T2 lesions. Bodini and colleagues showed that both the EDSS and the MSSS were not related to the tracer DVR in T2 lesions at a cross-sectional evaluation [15]. Physical disability was rather related to the extent of lesions demyelination over a follow-up period of four months [15]. We might speculate that physical disability is actually driven by the balance between demyelination and remyelination within MS lesion with a consequent axonal loss. Moreover, EDSS is a measure of physical disability with a low degree of specificity because it is mainly related to walking abilities, especially when it turns to the highest point. Timed 25-ft walk is actually more precise than EDSS and might explain the discrepancy in our finding.

In conclusion, our study reported that [^18^F]florbetapir PET imaging might be able to assess in vivo myelin pathology with a higher extent of specificity compared to MRI. The static SUV_70–90_ PET imaging evaluation provides similar information to the dynamic PET. Dynamic PET imaging requires long dynamic scans and, hence, high costs. Conversely, the SUV might be calculated through a static acquisition lasting for twenty minutes. This makes the technique highly appealing to implement in clinical settings. Clinicians could use the PET imaging as a tool to measure the subjective remyelinating potential, with the ultimate goal of personalizing treatment. They could also rely on its quantitative measures when assessing the effect of a drug targeting remyelination, e.g. opicinumab [50]. Finally, although further longitudinal evaluations are needed to test the reproducibility of the technique, measures derived from PET imaging (mean DVR or mean SUV) are able to measure the accumulation of tissue changes beyond the defined borders of the lesion.

## References

[1] Compston A, Coles A (2008). Multiple sclerosis. Lancet..

[2] Lublin FD, Reingold SC, Cohen JA, Cutter GR, Sorensen PS, Thompson AJ (2014). Defining the clinical course of multiple sclerosis: the 2013 revisions. Neurology..

[3] Ciccarelli O, Barkhof F, Bodini B, De Stefano N, Golay X, Nicolay K (2014). Pathogenesis of multiple sclerosis: insights from molecular and metabolic imaging. Lancet Neurol.

[4] Sahraian MA, Radue EW, Haller S, Kappos L (2010). Black holes in multiple sclerosis: definition, evolution, and clinical correlations. Acta Neurol Scand.

[5] Gallo A, Rovaris M, Riva R, Ghezzi A, Benedetti B, Martinelli V (2005). Diffusion-tensor magnetic resonance imaging detects normal-appearing white matter damage unrelated to short-term disease activity in patients at the earliest clinical stage of multiple sclerosis. Arch Neurol.

[6] Filippi M (2003). Magnetization transfer MRI in multiple sclerosis and other central nervous system disorders. Eur J Neurol.

[7] Niccolini F, Su P, Politis M (2015). PET in multiple sclerosis. Clin Nucl Med.

[8] Klunk WE, Engler H, Nordberg A, Wang Y, Blomqvist G, Holt DP (2004). Imaging brain amyloid in Alzheimer's disease with Pittsburgh compound-B. Ann Neurol.

[9] Price JC, Klunk WE, Lopresti BJ, Lu X, Hoge JA, Ziolko SK (2005). Kinetic modeling of amyloid binding in humans using PET imaging and Pittsburgh compound-B. J Cereb Blood Flow Metab.

[10] Reinke AA, Gestwicki JE (2011). Insight into amyloid structure using chemical probes. Chem Biol Drug Des.

[11] Bajaj A, LaPlante NE, Cotero VE, Fish KM, Bjerke RM, Siclovan T (2013). Identification of the protein target of myelin-binding ligands by immunohistochemistry and biochemical analyses. J Histochem Cytochem.

[12] Grecchi E, Veronese M, Bodini B, Garcia-Lorenzo D, Battaglini M, Stankoff B (2017). Multimodal partial volume correction: application to [(11)C]PIB PET/MRI myelin imaging in multiple sclerosis. J Cereb Blood Flow Metab.

[13] Biancalana M, Koide S (2010). Molecular mechanism of Thioflavin-T binding to amyloid fibrils. Biochim Biophys Acta.

[14] Matias-Guiu JA, Oreja-Guevara C, Cabrera-Martin MN, Moreno-Ramos T, Carreras JL, Matias-Guiu J (2016). Amyloid proteins and their role in multiple sclerosis. Considerations in the use of amyloid-PET imaging. Front Neurol.

[15] Bodini Benedetta, Veronese Mattia, García-Lorenzo Daniel, Battaglini Marco, Poirion Emilie, Chardain Audrey, Freeman Léorah, Louapre Céline, Tchikviladze Maya, Papeix Caroline, Dollé Frédéric, Zalc Bernard, Lubetzki Catherine, Bottlaender Michel, Turkheimer Federico, Stankoff Bruno (2016). Dynamic Imaging of Individual Remyelination Profiles in Multiple Sclerosis. Annals of Neurology.

[16] Polman CH, Reingold SC, Banwell B, Clanet M, Cohen JA, Filippi M (2011). Diagnostic criteria for multiple sclerosis: 2010 revisions to the McDonald criteria. Ann Neurol.

[17] Montgomery AJ, Thielemans K, Mehta MA, Turkheimer F, Mustafovic S, Grasby PM (2006). Correction of head movement on PET studies: comparison of methods. J Nucl Med.

[18] Ikoma Y, Edison P, Ramlackhansingh A, Brooks DJ, Turkheimer FE (2013). Reference region automatic extraction in dynamic [(11)C]PIB. J Cereb Blood Flow Metab.

[19] Logan J, Fowler JS, Volkow ND, Wang GJ, Ding YS, Alexoff DL (1996). Distribution volume ratios without blood sampling from graphical analysis of PET data. J Cereb Blood Flow Metab.

[20] Chard DT, Jackson JS, Miller DH, Wheeler-Kingshott CA (2010). Reducing the impact of white matter lesions on automated measures of brain gray and white matter volumes. J Magn Reson Imaging.

[21] Patenaude B, Smith SM, Kennedy DN, Jenkinson M (2011). A Bayesian model of shape and appearance for subcortical brain segmentation. Neuroimage..

[22] Heckemann RA, Keihaninejad S, Aljabar P, Rueckert D, Hajnal JV, Hammers A (2010). Improving intersubject image registration using tissue-class information benefits robustness and accuracy of multi-atlas based anatomical segmentation. Neuroimage..

[23] Smith SM, Zhang Y, Jenkinson M, Chen J, Matthews PM, Federico A (2002). Accurate, robust, and automated longitudinal and cross-sectional brain change analysis. Neuroimage..

[24] Behrens TE, Woolrich MW, Jenkinson M, Johansen-Berg H, Nunes RG, Clare S (2003). Characterization and propagation of uncertainty in diffusion-weighted MR imaging. Magn Reson Med.

[25] Andersson JL, Skare S, Ashburner J (2003). How to correct susceptibility distortions in spin-echo echo-planar images: application to diffusion tensor imaging. Neuroimage..

[26] Smith SM, Jenkinson M, Woolrich MW, Beckmann CF, Behrens TE, Johansen-Berg H (2004). Advances in functional and structural MR image analysis and implementation as FSL. Neuroimage..

[27] Jenkinson M, Bannister P, Brady M, Smith S (2002). Improved optimization for the robust and accurate linear registration and motion correction of brain images. Neuroimage..

[28] Alsop DC, Detre JA, Golay X, Gunther M, Hendrikse J, Hernandez-Garcia L (2015). Recommended implementation of arterial spin-labeled perfusion MRI for clinical applications: a consensus of the ISMRM perfusion study group and the European consortium for ASL in dementia. Magn Reson Med.

[29] van Walderveen MA, Kamphorst W, Scheltens P, van Waesberghe JH, Ravid R, Valk J (1998). Histopathologic correlate of hypointense lesions on T1-weighted spin-echo MRI in multiple sclerosis. Neurology..

[30] De Groot C. J. A. (2001). Post-mortem MRI-guided sampling of multiple sclerosis brain lesions: Increased yield of active demyelinating and (p)reactive lesions. Brain.

[31] Kutzelnigg A, Lucchinetti CF, Stadelmann C, Bruck W, Rauschka H, Bergmann M (2005). Cortical demyelination and diffuse white matter injury in multiple sclerosis. Brain..

[32] Schmierer K, Wheeler-Kingshott CA, Boulby PA, Scaravilli F, Altmann DR, Barker GJ (2007). Diffusion tensor imaging of post mortem multiple sclerosis brain. Neuroimage..

[33] Seewann A, Vrenken H, van der Valk P, Blezer EL, Knol DL, Castelijns JA (2009). Diffusely abnormal white matter in chronic multiple sclerosis: imaging and histopathologic analysis. Arch Neurol.

[34] Schmierer K, Scaravilli F, Altmann DR, Barker GJ, Miller DH (2004). Magnetization transfer ratio and myelin in postmortem multiple sclerosis brain. Ann Neurol.

[35] De Stefano N, Narayanan S, Francis SJ, Smith S, Mortilla M, Tartaglia MC (2002). Diffuse axonal and tissue injury in patients with multiple sclerosis with low cerebral lesion load and no disability. Arch Neurol.

[36] Lucchinetti CF, Popescu BF, Bunyan RF, Moll NM, Roemer SF, Lassmann H (2011). Inflammatory cortical demyelination in early multiple sclerosis. N Engl J Med.

[37] Giannetti P, Politis M, Su P, Turkheimer FE, Malik O, Keihaninejad S (2015). Increased PK11195-PET binding in normal-appearing white matter in clinically isolated syndrome. Brain..

[38] Elliott Colm, Wolinsky Jerry S, Hauser Stephen L, Kappos Ludwig, Barkhof Frederik, Bernasconi Corrado, Wei Wei, Belachew Shibeshih, Arnold Douglas L (2018). Slowly expanding/evolving lesions as a magnetic resonance imaging marker of chronic active multiple sclerosis lesions. Multiple Sclerosis Journal.

[39] Bullich S, Villemagne VL, Catafau AM, Jovalekic A, Koglin N, Rowe CC (2017). Optimal reference region to measure longitudinal amyloid-beta change with (18)F-Florbetaben PET. J Nucl Med.

[40] Scott G, Ramlackhansingh AF, Edison P, Hellyer P, Cole J, Veronese M (2016). Amyloid pathology and axonal injury after brain trauma. Neurology..

[41] Fragoso YD, Wille PR, Abreu M, Brooks JBB, Dias RM, Duarte JA (2017). Correlation of clinical findings and brain volume data in multiple sclerosis. J Clin Neurosci.

[42] De Stefano N, Matthews PM, Filippi M, Agosta F, De Luca M, Bartolozzi ML (2003). Evidence of early cortical atrophy in MS: relevance to white matter changes and disability. Neurology..

[43] Sepulcre J, Goni J, Masdeu JC, Bejarano B, Velez de Mendizabal N, Toledo JB (2009). Contribution of white matter lesions to gray matter atrophy in multiple sclerosis: evidence from voxel-based analysis of T1 lesions in the visual pathway. Arch Neurol.

[44] Freeman L, Garcia-Lorenzo D, Bottin L, Leroy C, Louapre C, Bodini B (2015). The neuronal component of gray matter damage in multiple sclerosis: a [(11) C]flumazenil positron emission tomography study. Ann Neurol.

[45] You Y, Joseph C, Wang C, Gupta V, Liu S, Yiannikas C (2019). Demyelination precedes axonal loss in the transneuronal spread of human neurodegenerative disease. Brain..

[46] Ge Y, Law M, Johnson G, Herbert J, Babb JS, Mannon LJ (2005). Dynamic susceptibility contrast perfusion MR imaging of multiple sclerosis lesions: characterizing hemodynamic impairment and inflammatory activity. AJNR Am J Neuroradiol.

[47] Narayana PA, Zhou Y, Hasan KM, Datta S, Sun X, Wolinsky JS (2014). Hypoperfusion and T1-hypointense lesions in white matter in multiple sclerosis. Mult Scler.

[48] D'Haeseleer M, Beelen R, Fierens Y, Cambron M, Vanbinst AM, Verborgh C (2013). Cerebral hypoperfusion in multiple sclerosis is reversible and mediated by endothelin-1. Proc Natl Acad Sci U S A.

[49] van Berckel BN, Ossenkoppele R, Tolboom N, Yaqub M, Foster-Dingley JC, Windhorst AD (2013). Longitudinal amyloid imaging using 11C-PiB: methodologic considerations. J Nucl Med.

[50] Ruggieri S, Tortorella C, Gasperini C (2017). Anti lingo 1 (opicinumab) a new monoclonal antibody tested in relapsing remitting multiple sclerosis. Expert Rev Neurother.

